# As above, so below? The influence of leader humor on bootleg innovation: The mechanism of psychological empowerment and affective trust in leaders

**DOI:** 10.3389/fpsyg.2022.956782

**Published:** 2022-09-16

**Authors:** Xiong Zheng, Sheng Mai, Chunguang Zhou, Liang Ma, Xiaomeng Sun

**Affiliations:** ^1^School of Economics and Management, Shihezi University, Shihezi, China; ^2^Graduate School, La Consolacion University Philippines, Malolos, Philippines; ^3^Normal College, Shihezi University, Shihezi, China

**Keywords:** leader humor, bootleg innovation, psychological empowerment, trust in leader, benign violation theory

## Abstract

Leadership humor is widely used in management practice and has aroused extensive discussion in academia. On account of the two-sided influence of leader humor on employees, its double-edged sword effect on employee behavior has been put more emphasis. As a benign violation of organizational norms and a kind of pro-organizational violation, respectively, both Leadership humor and employee bootleg innovation have the characteristics of violating organizational norms, but few studies have examined the relationship between them. Based on benign violation theory and social cognition theory, this study conducted a two-stage questionnaire survey and statistical-econometric analysis of 324 employees in 23 IT and manufacturing technology companies in Guangdong, Jiangsu, Zhejiang, Hubei, Beijing, and Shanghai, China. It not only examined the relationship between leadership humor and employee bootleg innovation but also tested a moderated mediation model. Results show that leadership humor is positively correlated with psychological empowerment and employee bootleg innovation, namely, leadership humor indirectly and positively affects employee bootleg innovation through psychological empowerment. Moreover, the indirect effect is positively regulated by leadership emotional trust. The higher the emotional trust of employees to leadership, the stronger the mediating role of psychological empowerment between leader humor and bootleg innovation behavior. The study enriches the existing path of research on the double-edged impact of leader humor on employee behavior, expands the boundary conditions of the relationship between empowered cognitive mediation and employee bootleg innovation and provides enlightenments for Chinese leaders to effectively apply the tool of leader humor.

## Introduction

As an effective tool of management practice (Malone, 1980; Mao et al., 2017), leader humor plays an important role in leadership effectiveness (Kong et al., 2019). Leader humor significantly predicts outcomes such as employee performance (Jing and Zhou, 2019; Wu et al., 2020), organizational citizenship behavior (Cooper et al., 2018; Rosenberg et al., 2021), and work engagement (Yam et al., 2018; Peng et al., 2019). Thus, many successful leaders apply humor to practical social communication and business activities. Many western political and business organizations also use humor as an important element of leadership training to improve leader effectiveness (Neves and Karagonlar, 2020; Chen et al., 2021). On the contrary, in traditional Chinese work values, work is serious. Moreover, leader humor at work is often regarded by subordinates as a kind of cheeky and improper behavior that cannot reflect the majesty of leaders. Thus, humor management research has been greatly neglected in the field of Chinese domestic management for a long time. However, as the new generation employees gradually step into the center of the work arena, their work values are significantly different from those of Chinese tradition. They reject the traditional hierarchical management concept and the serious and authoritative work atmosphere. Moreover, they pursue the work atmosphere of equality, empowerment, and fun and enjoy leader humor. Therefore, Chinese enterprise leaders are paying more attention to humor and are using this management tool. At present, theoretical research on local leader humor is still relatively scarce and lacks attention. Most studies have focus on review research, and empirical research needs to be enriched (Peng et al., 2019). Insufficient research on the topic of leader humor in China leads to a lack of theoretical references for entrepreneurs when applying the management tool.

Leader humor was a kind of leadership trait in which leaders used humorous language and behavior to entertain their subordinates in the interaction with them (Mao et al., 2017). Academic exploration of the positive aspects of leader humor included employee performance, attitudes, emotions, behaviors (Jing and Zhou, 2019; Kong et al., 2019). Recently, Yam et al. (2018) and Tang and Sun (2021) found that leader humor not only stimulates innovative behavior but also leads to deviant behavior. And bootleg innovation is the spontaneous innovation conducted in a deviant manner without the formal support of the organization, which is carried out by employees to improve organizational benefits (Criscuolo et al., 2014). In addition, bootleg innovation is a combination of innovative and deviant behavior (Jiang, 2018). Thus, can leader humor trigger bootleg innovation behavior in employees? Further empirical research is needed to provide a clear answer (Jing and Zhou, 2019). And our study supposes that leaders humor is a predictor of employee bootleg innovation.

Leader humor is regarded as an empowerment management strategy that initiates the empowerment cognition of subordinate employees (Gkorezis et al., 2011; Wang and Yang, 2019; Ali et al., 2021). The benign violation theory (BVT) is often used to explain two conditions for humor. Therefore, the creation of humor indicates the generation of benign violations. One is the existence of a violation of expected organizational norms, and the other is a benign violation (Mcgraw and Warren, 2010; Shi et al., 2021). According to the social cognitive theory (SCT), it is determined by the interaction of environment, individual cognition, and individual behavior (Bandura, 1986). In a highly contextualized organizational environment, the leader’s behavior is perceived by employees as the right way to do things (Shi et al., 2021). That is, in specific organizational contexts, leader humor is presented by a variety of benign ways of violating organizational norms (Yam et al., 2018). By integrating these two theories (BVT and SCT), we believe that the generation of leadership humor means the permission of leaders and organizations to benign violations, more specifically, an empowerment to employees’ psychological cognition, especially in the form of humor and jokes. In other words, leader humor brings empowering experiences to employees as manifested by the high intrinsic motivation of employees, and employees will also imitate and learn the benign violation behaviors behind leader humor and finally trigger the prosocial bootleg behaviors (Yam et al., 2018; Yuan and Zhang, 2021), such as bootlegging (Jia and Liu, 2021). Therefore, our study assumes that psychological empowerment mediates the process of leader humor influences bootlegging.

As an empowering management form, leader humor can inspire a sense of empowerment within employees. Then, the strength of the ultimate effect of leader humor on the behavior of subordinates is related to the interpersonal environment in which the employees live (Decker, 1987). Previous research has identified trust as a key interpersonal contextual factor influencing subordinates’ assessment of leader humor (Mayer et al., 1995; Masih et al., 2022). Bootlegging is a high-risk activity to achieve innovation in a deviant way, and employees effectively assess the risks behind it before deciding whether to implement it. Employees with a high sense of leadership trust believe that they would be understood and supported by their leaders when they innovate in a deviant way for the benefit of the organization. They also believe that leaders put themselves in the shoes of them and care about their welfare, thereby reducing the perception that they may be punished by their leaders for deviant innovation (Shockley-Zalabak et al., 2000). Thus, the mediating role of employee psychological empowerment may vary with the degree of trust that employees have in their leaders. As leader humor is a leadership style in terms of interpersonal communication, the emotional trust of leaders may play a moderating role in psychological empowerment to influence bootlegging. Furthermore, our study supposes the emotional trust of leaders moderates the mediating role of psychological empowerment between leadership humor and bootlegging.

To sum up, based on previous studies on leader humor and bootleg innovation, by integrating BVT and SCT, we discuss whether and how leadership humor affects employee bootleg innovation. Specifically, we explored the mediating role of psychological empowerment in the influence of leadership humor on bootleg innovation and the moderating role of leadership emotional trust in this process.

The contributions of this study are as follows. First, this study enriches the variables of leader humor research in the Chinese context and responds to focus on the two-sided role of leader humor in influencing effects (Jing and Zhou, 2019). This study integrates the duality of leader humor to inform future research on the complexity of leader humor, such as pro-organizational non-ethical behavior, constructive bootleg behavior, and coercive organizational citizenship behavior. Second, this study expands the role mechanism of the influence of leader humor from the perspective of motivation. The study also uses psychological empowerment as a mediating variable to explore the role path of leader humor on bootlegging behaviors. This study makes up for the shortage of previous research perspectives and can provide a new path for later research. Finally, this study complements the boundary conditions of the effect of leader humor. It also explores the conditions of the mediating process of “leader humor–psychological empowerment–employee bootleg innovation” based on interpersonal interactions and the mediating path of negative regulation of leader emotional trust. Moreover, this study enriches the boundary conditions of the effect of leader humor at the interaction level between leaders and direct subordinates. The innovation points are as follows. First, this study enriches the existing path of research on the impact of leader humor on employee behavior from the perspective of cognitive empowerment. Second, the study expands the boundary conditions of the relationship between empowered cognitive mediation and employee bootleg innovation from the perspective of interpersonal trust relationships. This study also provides t theoretical guidance for the local management practice of leader humor. Especially with the repeated occurrence of the COVID-19, employees’ mental health and work initiative have been seriously damaged, and employees’ trust in the organization and leaders has also been negatively affected (Hu et al., 2020). This study believes that leadership humor, as a good management tool, can significantly smooth the negative impact of COVID-19. Specifically, leadership humor as a kind of authorization work, It can help improve employees’ positive psychological state and improve their initiative deviant innovation behavior.

The remainder of this study is further organized as follows. First, we review the literature on leader humor, identify research gaps from existing studies and confirm the relationship between leader humor and bootlegging. Then, we propose the hypotheses of the mediating roles of psychological empowerment and leader emotional trust. Second, we describe the research methods, data collection, and measurement instruments. Third, we test the theoretical hypotheses of this study, obtain their validation results, and further discuss their empirical results. Finally, we describe the final conclusions of this study and management insights.

## Theoretical background and hypothesis development

### Leader humor and bootlegging

Bootlegging is the spontaneous innovation conducted in a deviant manner without the formal support of management, which is carried out by grassroots employees to improve organizational benefits (Criscuolo et al., 2014). Bootlegging is considered to have the dual properties of prosocial and antisocial, including a combination of innovative and deviant behavior (Jiang, 2018). Bootlegging is a special kind of constructive deviant behavior by definition. For example, employees secretly improve their work methods privately and against the instructions of their superiors to enhance the organization’s individual creative performance and organizational interests, which is also an extra role behavior that violates organizational norms (Wang and Wang, 2020). Early studies focused on the influencing factors of employee bootleg innovation from the perspective of individual employees and organizations. Later, some scholars believed that leader was also an important factor influencing employee bootleg innovation (Criscuolo et al., 2014; Jiang, 2018), such as leadership style and leadership behavior.

Supervisors use humorous language styles in their daily interactions with subordinate employees, using their own humorous traits to entertain subordinates, which can have a significant impact on employees’ work behavior (Jing and Zhou, 2019; Huang, 2022). According to the BVT, the creation of leader humor indicates the generation of benign conflict behavior (Mcgraw and Warren, 2010). Given that humor often requires violating norms, leader humor sends a signal to the surrounding subordinates that some violating norms are permissible in interpersonal interactions with subordinates and superiors. According to the social cognitive theory (SCT), it is determined by the interaction of environment, individual cognition, and individual behavior (Bandura, 1986). In a highly contextualized organizational environment, the leader’s behavior is perceived by employees as the right way to do things. The leader’s behavior is a role model and a symbol of organizational norms, which leads subordinate employees to understand the surroundings and act with the organization’s norms and expectations (Salancik and Pfeffer, 1978; Shamir et al., 1993). Employees used their own initiative to receive and understand information about the organizational environment (particularly from leadership sources) by observing leadership behavior and learning to imitate it (Bandura, 1986). The leader’s behavior and interpersonal style can send powerful messages to employees when employees receive these messages knowing which behaviors are accepted, encouraged, and punished by the organization. And the leader’s humor encourages the occurrence of benign violations of organizational norms.

According to the BVT (Mcgraw and Warren, 2010) and SCT (Bandura, 1986), leader humor is a demonstration of benign norm violation (Yam et al., 2018), and the leader’s behavior is perceived by employees as the right way to do things (Shi et al., 2021). When leaders humorously communicate with their employees, owing to their special, exemplary position in the organization, employees will receive, learn, and imitate the benign violation messages, which ultimately influence their own behaviors. Specifically, leader humor increases employees’ imitative motivation to engage in benign behavior violation of organizational norms and creates a relaxed and enjoyable work environment that promotes innovation (Tang and Sun, 2021). Leader humor conveys to employees that violating organizational norms may be permissible when their creative ideas and novelty are not formally recognized by the organization (Yam et al., 2018). Thus, employees may practice their creativity by imitating benign behavior and *via* bootleg innovation. The reason is that they believe that bootlegging may be acceptable and supported by their leaders in this issue, and they will not be punished. They also believe that leaders have authorized their bootleg innovation. We thus hypothesize:

*Hypothesis 1: Leader humor is positively related to employee bootleg innovation*.

### Mediating role of psychological empowerment

Psychological empowerment is the mental state being experienced by individuals. It is an intrinsic task motivation manifested as four cognitions, namely, meaning, self-efficacy, self-determination, impact (Spreitzer, 1995). Spreitzer (1995) noted that meaning refers to the value of work goals judged by individuals according to their own ideal standards. Self-efficacy is a perception of ability and the belief that an individual is capable of doing the job. Then, self-determination is the discretion of work methods and investment decisions. Moreover, impact refers to the degree to which an individual can influence organizational strategy, organizational management, or organizational performance at work. Many researches have focused on the key mediating role that psychological empowerment plays a role between leadership style and employee attitudes, emotions, and behaviors (Chen et al., 2019; Jing and Zhou, 2019; Rosenberg et al., 2021). Existing research has confirmed that leader humor is a successful empowerment management practice that effectively stimulates employees’ perceptions of empowerment, influencing their work attitudes and behaviors (Gkorezis et al., 2011; Ali et al., 2021).

When leaders interact with employees in a humorous way, employees receive the message and form a positive perception that their performance is recognized and praised by the leader and is considered an external motivation (Crawford, 1994; Meyer, 2000; Billig, 2005). According to the SCT, employees’ internal imitation motivation is inspired by the external environment, and their positive cognitions of their work and abilities are expressed as high intrinsic work motivation (Crawford, 1994) and competence (Bandura, 1986). This notion is a psychological perception formed by employees based on considerable information in a special organizational environment, and this perception can be regarded as a kind of empowerment. Thus, as an empowerment management strategy, leader humor can remove the interpersonal obstacles associated with formal hierarchies in the workplace and increase the sense of empowerment of subordinates (Gkorezis et al., 2011; Jia and Liu, 2021). When leaders and employees communicate with humor, this case not only makes the work more meaningful (Duncan, 1982) but also enhances employees’ work autonomy, self-efficacy and self-confidence (Jiang et al., 2020; Ali et al., 2021), thereby improving employees’ perception of impact on the job. The sense of work meaning, self-efficacy, self-determination, and work impact experienced by subordinates is exactly the four sub-dimensions of psychological empowerment. We thus hypothesize:

*Hypothesis 2: Leader humor is positively related to employee psychological empowerment*.

As a form of empowerment management, leader humor can effectively arouse employees’ psychological empowerment perceptions (Ali et al., 2021). In addition, high perception, in turn, positively influences employees’ prosocial violations (Yan et al., 2017). Empowerment implies that employees can break some rules to innovate. Bootlegging, a typical prosocial violation, might be significantly predicted by psychological empowerment.

Specifically, employees’ high sense of work indicates their recognition of work value and inspires a sense of ownership. When ideas are not affirmed, employees have an incentive to continue to implement innovative behaviors in ways that violate organizational norms to advance the future interests of the organization (Jiang, 2018). Employees’ high self-efficacy perception indicates confidence in their ability to complete the job. Such employees are prone to innovation and consistently practice creative ideas, providing psychological motivation for bootlegging (Yao and Han, 2013). A high work autonomy means that employees are highly selective in their own working methods and efforts. When their ideas are not supported, employees also continue to be able to act according to their own wishes and methods (Liu and Zou, 2013). They continue to innovate privately by violating organizational norms to accomplish tasks without official permission (Mcgraw and Warren, 2010). Employees with a high perception of work influence believe that their behavior has an important role in achieving organizational goals, which also provides cognitive resources for bootlegging. When employees are unable to innovate in the proper way, high perception dispels concerns about bootlegging and makes them take risks to innovate privately to enhance the organization’s interests. Therefore, psychological empowerment has a significant positive impact on bootlegging.

According to the BVT and SCT, humor is essentially a benign violation. Thus, as a slight violation of organizational norms, leader humor sends a signal to employees, which can be regarded as empowerment (Ali et al., 2021). Performing benign violations of organizational norms is accepted and encouraged. When employees perceive empowerment (Gkorezis et al., 2011), they learn to imitate the benign violation characteristic behind leader humor, which in turn stimulates their prosocial deviant behavior (Yan et al., 2017; Jia and Liu, 2021). For example, when employees’ ideas are not recognized, the humorous perception causes the employees to continue to implement their ideas for the benefit of the organization and creates bootlegging. We thus hypothesize:

*Hypothesis 3: Leader humor is positively related to bootlegging mediated by increased psychological empowerment*.

### Moderating effect of leadership emotional trust

Leadership emotional trust refers to the emotional trust of employees in their direct leaders. It is the emotional bond and interpersonal care between subordinates and their direct superiors. Leadership emotional trust seems to pay attention to each other’s wellbeing and fully consider each other’s interests (Mcallister, 1995). Leadership trust significantly influences employees’ understanding of interpersonal relationships between superiors and subordinates (Mayer et al., 1995; Masih et al., 2022). Leadership trust is a key situational factor to describe the interpersonal relationship between leaders and subordinates (Islam et al., 2021). Therefore, the effectiveness of leader humor depends on employees’ reactions and interpretations of humor motives, that is, the impact of leader humor on employees’ ultimate behavior may vary with the level of employee’s trust in the leader (Peng et al., 2019; Wang and Yang, 2019; Masih et al., 2022).

Although no scholars have directly studied the moderating role of leadership emotional trust in leader humor and employee behavior. But there are some studies using trust in leader as a moderator of leaders’ humor and employees’ behaviors (Rosenberg et al., 2021; Masih et al., 2022). Kim et al. (2016) believed that subordinates’ trust in their direct superiors is a key situation for employees’ behavioral responses after cognitive evaluation of leader humor, particularly the dimension of leadership emotional trust. When employees have high emotional trust in their direct supervisors, the emotional bond between them is also stronger, making them take better care of each other (Yao and Han, 2013; Tremblay, 2017). Therefore, these employees are more likely to consider themselves and leaders as a community of interest linked together to achieve organizational goals (Gong et al., 2013). This difference in emotional bonding based on different trust relationships could influence employees’ behaviors beyond their roles after they perceive empowerment. Wan (2009) found that the interaction between employee psychological empowerment and leadership trust has a significant positive effect on employees’ organizational citizenship behavior. The sense of leadership trust encourages employees to engage in pro-organizational extra-role behaviors after perceiving psychological empowerment, such as bootlegging. Therefore, this study concludes that employees with high leadership emotional trust are more likely to transform this empowerment cognition into bootlegging. Leadership emotional trust positively moderates the effect of psychological empowerment on bootlegging.

Specifically, as a motivational empowerment signal, leader humor stimulates employees’ high perception of empowerment. Whether this sense transforms into bootlegging depends on the employees’ risk assessment behind deviant innovation. Compared with employees with low emotional trust, those with high leadership emotional trust are more likely to deal with the empowerment signal of leader humor (Shockley-Zalabak et al., 2000; Gkorezis and Bellou, 2016; Rosenberg et al., 2021). They are more likely to believe that their leaders are on their side. They are in the long-term interest of the organization and are unlikely to be punished by leaders even if they innovate in a bootleg manner (Wan, 2009). This perception of psychological safety triggered by high leadership emotional trust reduces employees’ risk assessment behind bootlegging, thereby making them more likely to engage in bootleg innovative behavior. We thus hypothesize:

*Hypothesis 4: Leadership emotional trust positively moderates the relationship between psychological empowerment and bootlegging, such that the relationship between psychological empowerment and bootlegging becomes stronger as the leadership emotional trust increases*.

*Hypothesis 5: Leadership emotional trust positively moderates the mediating role of psychological empowerment between leader humor and bootlegging. The higher the level of leader emotional trust, the stronger the mediating role psychological empowerment plays between leader humor and bootlegging, and vice versa*.

**FIGURE 1:**
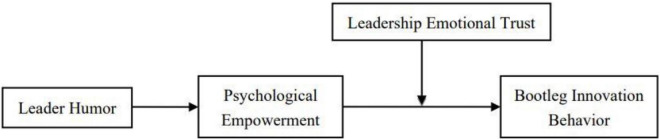
Research theoretical model.

According to the above Hypotheses, Figure 1 shows the theoretical model of this study.

## Methodology

Based on a questionnaire survey and SPSS 26.0, this study used to perform reliability and discriminant validity tests, descriptive statistics, correlation analysis, and regression analysis. This study used hierarchical regression and the Bootstrapping method in the SPSS-PROCESS program model 7 to test for mediating effects and mediating effects with moderation. To further clearly demonstrate the mediating effect with moderation, previous studies usually made a plot of the moderating effect with a simple slope of one standard deviation of the moderating variable. However, the method of grouping moderating variables can only test the difference of indirect effects under two values of leader emotional trust. Thus, we cannot show the full process of the effect of the continuous variable of moderating variables on indirect effects.

### Data sources

We contacted 32 part-time Master of Business Administration (MBA) students to help us to collect the survey data in this research. All students worked full-time and were enrolled in a MBA program part-time at a large university in China, and All students were the middle or senior managers in their company. With the help of the students, we invited employees of their companies to participate in our research. And the study sample was drawn from 23 IT and manufacturing technology companies in Guangdong, Jiangsu, Zhejiang, Hubei, Beijing, and Shanghai, China. The employees who filled out the questionnaire were mainly from research and development (R&D), human resources (HR), marketing, and other departments with high autonomy and innovative work characteristics. The questionnaires were mainly distributed to the new generation (born in 1980–1999) of employees. The survey period was from July to September 2021. Owing to the implicit nature of bootlegging, the research adopted the way of employee self-completion. Questionnaires were distributed online and offline according to the regional differences of the sample, including email, questionnaire star, and paper distribution. And In addition, to control the homologation method bias, the questionnaire survey was divided into two stages in time. In the first stage, the demographic variables of employees, leader humor, psychological empowerment, and leadership emotional trust were investigated. The second stage of bootlegging survey was conducted 1 month later. The questionnaires were matched by two methods of pre-numbering and personal contact information for a complete questionnaire. A total of 450 questionnaires (online accounting for 44.0%) was distributed in two stages, and all of them were collected in the first stage and 346 in the second stage. The 324 valid questionnaires (online accounting for 37.0%) were integrated and paired, with a valid recovery rate of 72.0%. Among them, in terms of gender, 46.6% w ere female, and 53.4% were male, which was a reasonable ratio between men and women. In terms of age, 6.8% of employees were born before 1979, 36.7% born in 1980–1989, 54.0% born in 1990–1999, and 2.5% born after 2000. In terms of education level, there were six employees with college or below, 199 employees with a bachelor’s degree, 85 employees with a master’s degree, and 34 employees with a doctorate, accounting for 1.9, 61.4, 26.2, and 10.5%, respectively. R&D personnel accounted for 72.2%, marketing personnel accounted for 24.7%, and other departments, such as HR, accounted for 3.1%.

Since the variables are measured in the form of employee self-reports, issues of common method bias may exist. This study was tested using the Harman one-way test. By analyzing the topic, we found seven principal component factors with eigenvalues greater than one, and the cumulative variance was 83.44%. The first factor explained 36.05% of the variance, accounting for less than half of the total variance. Then, the latent variable model control method was used to test CMV. The fitting results are shown in the last row of Table 1. The latent variable model including CMV is compared with the 4-factor model, χ ^2^ / D F, RMSEA, CFI and other indicators change little, indicating that the latent variable model containing CM- V is not significantly better than the original 4-factor model. Therefore, using Harman one-way test and latent variable model control method to test the CMV, and it can mutually verify that the CMV of the scale is within the acceptable range.

**TABLE 1:** Results of validated factor analysis of the discriminant validity of variables (*N* = 324).

Model	Factor	χ^2^/*df*	RMSEA	GFI	CFI	TLI	IFI
Four-factor model	A, B, C, D	2.173	0.060	0.855	0.960	0.954	0.960
Three-factor model	A + B, C, D	9.844	0.165	0.540	0.681	0.653	0.682
Two-factor model	A + B, C + D	13.645	0.198	0.414	0.541	0.505	0.542
One-factor model	A + B + C + D	14.988	0.208	0.374	0.491	0.452	0.492
Five-factor model	A, B, C, D, CMV	2.168	0.059	0.857	0.961	0.956	0.961

A, B, C, and D stand for leader humor, psychological empowerment, leadership emotional trust, and bootleg innovation, respectively; + indicates factor merging.

### Variables and measures

The mature scale was used in this study, and all questions in this study were Likert seven-point scale, except for the control variables. The details of the variables were as follows.

#### Leader humor

The seven-point scale developed by Yam et al. (2018) was used. We communicated with the author *via* email to obtain the Chinese version of this scale, with sample questions such as “My leader speaks in a humorous way that makes people laugh.”

#### Bootlegging

The five-point scale developed by Criscuolo et al. (2014) was used. Examples include “I like to think of new ideas outside of the main work.”

#### Psychological empowerment

The 12-point sample scale was developed based on Spreitzer (1995), with the sample question “The work I do is very meaningful.”

#### Leadership emotional trust

The emotional trust dimension of the trust scale developed by Mcallister (1995) was used. The scale has five items, and the sample question includes “During the contact with the leader, I can communicate my thoughts and feelings with him without any constraint.”

#### Control variables

Demographic variables, such as employees’ gender, age, education, and time spent with superiors and subordinates, had significant effects on bootlegging (Criscuolo et al., 2014; Jiang, 2018). Thus, these variables were controlled in this study.

### Reliability and discriminant validity tests

The Cronbach’s α coefficients for leader humor, bootleg innovation, psychological empowerment, and emotional trust in leadership are 0.865, 0.901, 0.903, and 0.924, respectively. These figures are all within the confidence interval of greater than 0.7, with good reliability. To test the discriminant validity among the variables, this study was conducted using validated factor analysis. In Table 1, all data fit indicators of the four-factor model were significantly better than the other models (χ^2^ = 773.660, *df* = 356, χ^2^/*df* = 2.173, CFI = 0.960, TLI = 0.954, IFI = 0.960, RMSEA = 0.060), indicating that the four core variables in this study had good discriminant validity (Becker, 2005).

## Results analysis

### Summary statistics and correlation analysis

Table 2 presents the means, standard deviations, and correlation coefficients of the main variables. Leader humor is positively related to psychological empowerment and bootlegging (*r* = 0.556, *p* < 0.01; *r* = 0.443, *p* < 0.01), and psychological empowerment is positively related to bootlegging (*r* = 0.466, *p* < 0.01), supporting H1 and H2.

**TABLE 2:** Means, standard deviations, and pearson correlation coefficients of the main variables.

Variables	Means	SD	1	2	3	4
1. Leader humor	3.84	1.41				
2. Psychological empowerment	4.52	1.08	0.556**			
3. Leadership emotional trust	4.11	1.05	0.043	0.035		
4. Bootleg innovation	4.07	1.02	0.443**	0.466**	0.369**	

This table does not contain control variables; **p < 0.01.

### Hypothesis test

We standardize the data before regression. As standardization contains centrality, standardization reduces multicollinearity among variables.

#### Tests for the main and mediating effects

Table 3 presents the regression results. From Models 2 and 4, leader humor has a significant positive effect on bootlegging (β = 0.437, *p* < 0.001) and psychological empowerment (β = 0.558, *p* < 0.001), evidenced by H1 and H2. In Model 5, the coefficient of the effect of leader humor on bootlegging becomes smaller and it is still significant significant (β = 0.260, *p* < 0.001) after putting leader humor and psychological empowerment into the regression equation. Thus, H3 is supported.

**TABLE 3:** Results of the hierarchical regression model.

Variables	Psychological empowerment			Bootleg innovation behavior		
	Model 1	Model 2	Model 3	Model 4	Model 5	Model 6
Gender	0.037	−0.030	0.150**	0.097	0.107*	0.117**
Age	0.053	0.021	0.035	0.009	0.003	−0.020
Education	0.087	−0.058	0.040	0.017	−0.001	0.014
Time	−0.019	−0.046	−0.101	−0.121*	−0.107*	–0.084
Leader humor		0.558***		0.437***	0.260***	0.258***
Psychological empowerment					0.316***	0.301***
Leadership emotional trust						0.346***
PE × LET						0.135**
Δ*R*^2^		0.306***		0.007***	0.067***	0.137***
F	1.925	29.269***	2.689***	17.816***	21.296***	26.219***

*p < 0.05, **p < 0.01, ***p < 0.001.

#### Robustness test of the intermediation effect

Meanwhile, this study uses the Bootstrap method to further verify the mediating role of psychological empowerment to improve the robustness of the results. Table 4 shows the results of the Bootstrap analysis of the mediating effect with 95% confidence intervals using the PROCESS plug-in program. The mediating effect value of leader humor influencing bootlegging through psychological empowerment is 0.177 (CI = [0.153, 0.442]), without containing zero. H3 is again confirmed.

**TABLE 4:** Results of robustness tests of the intermediary mechanism.

Dependent variable	Effect type	Effect value	Standard error	95% CI		Effect ratio
				Upper	Lower	
Bootleg innovation behavior	Indirect	0.177	0.058	0.153	0.442	40.50%
	Direct	0.260	0.040	0.098	0.256	59.50%
	Total	0.437	0.050	0.339	0.474	100%

#### Moderating effect of leadership emotional trust

In Model 6 of Table 3, the interaction term between psychological empowerment and leadership emotional trust has a significant positive effect on bootlegging (β = 0.437, *p* < 0.01). This result indicates that leadership emotional trust positively moderates the impact of psychological empowerment on bootlegging. H4 is supported.

In this study, the Bootstrap method is used to further test the mediating effect with moderation. In one standard deviation grouping criterion for differences, the direct and indirect effects of leader humor and bootlegging are described separately in high and low levels of leadership emotional trust. Table 5 shows the results. The difference in indirect effects between high and low leader emotional trust is significantly positive (β = 0.150, and CI = [0.153, 0.442] and without containing zero). Thus, leadership emotional trust positively moderates the mediating role of psychological empowerment between leader humor and bootlegging, evidenced by H5.

**TABLE 5:** Results of tests with moderated mediating effects.

Effect type	Adjustment variable	Effect value	Standard error	95% CI lower	95% CI upper
Indirect	High leadership emotional trust	0.243	0.052	0.148	0.352
	Low leadership emotional trust	0.093	0.044	0.005	0.178
	Difference	0.150	0.061	0.039	0.276

**FIGURE 2:**
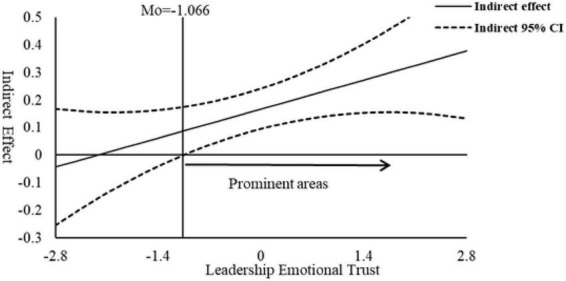
Test for mediating effects with moderation.

This study Referring to the practice of Preacher et al. (2007), uses the J-N method to make a moderated mediation effect map of 95% confidence bands and specific significant regions. Figure 2 shows the results. The straight line represents the linear correlation of the indirect effect on the moderating variable, and the dotted line area represents the 95% confidence interval band. When the relative value of leadership emotion trust is greater than −1.066 (standardized), the significant area shows that the indirect effect of leader humor on bootlegging through psychological empowerment holds significantly.

## Discussion

### Theoretical discussion

Leaders humor not only stimulates innovative behavior (Tang and Sun, 2021), but also leads to deviant behavior (Yam et al., 2018; Shi et al., 2021). Bootleg innovation is the combination of these two behaviors. Will a leader’s humor trigger employees’ Bootleg innovation? To answers this question, we use the survey study to explore that whether and how leadership humor affects employees’ bootleg innovation. Here are our findings. Leader humor has a significant positive effect on employee bootleg innovation. And psychological empowerment plays an intermediary role between leader humor and bootleg innovation behavior. In an addition leadership emotional trust positively moderates the mediating role of psychological empowerment between leader humor and bootleg innovation behavior.

First, is the impact of leader humor always positive? The literature suggested that leader humor had not only a positive impact on employees but also some negative effects. A significant double-edged effect exists (Yam et al., 2018). Tang and Sun (2021) found that leader humor was conducive to employee innovation. However, Yam et al. (2018) and Shi et al. (2021) showed that leader humor promoted bootlegging. Hence, scholars called for the need to conduct research on the double-edged impact of leader humor, particularly the need to include both sides of the impact of leader humor in the same outcome variable (Jing and Zhou, 2019). Examples include verifying the impact of leader humor on pro-organizational non-ethical behavior, constructive bootleg behavior, and bootlegging. In response to existing research, this study explores the double-edged role of leader humor by including bootlegging as an outcome variable. The results show that the higher the level of leader humor, the higher the level of bootlegging. Therefore, leader humor has not only a positive side but also a negative side, and its resultant variables have the same dual property of benign violation. This study integrates the double-edged nature of leader humor in influencing employee behavior, enriches the outcome variables of leader humor research in China, and responds to recent calls to focus on the double-edged role (Jing and Zhou, 2019). Furthermore, this study provides lessons for future research on the complexity of leader humor influence themes.

Second, leader humor is seen as an empowering management strategy (Ali et al., 2021). Can this perception of empowerment transform into bootlegging? The higher the level of leader humor, the higher the level of employees’ perception of psychological empowerment (Gkorezis et al., 2011). In addition, the level of employees’ pro-organizational violations is higher (Yan et al., 2017; Yuan and Zhang, 2021), such as bootlegging (Jia and Liu, 2021). The results indicate that leader humor positively influences psychological empowerment, and psychological empowerment positively influences bootlegging. Psychological empowerment partially mediates the relationship. This high perception of empowerment is the cognitive basis for employees to engage in bootlegging. The study expands the mechanism of the effect and explores the path of the effect using psychological empowerment as a mediating variable to make up for the shortcomings of previous research perspectives.

Third, the effect of leader humor is related to the interpersonal environment in which the employee is located (Decker, 1987). However, is the mediating role of psychological empowerment influenced by the emotional trust of the leader? Leader humor brings a perception of empowerment to employees (Ali et al., 2021). However, the employees who are at low emotional trust would be concerned about the high risk behind bootlegging. Although leader humor gives them a high sense of empowerment, they are still less likely to risk bootlegging. For employees with high emotional trust, the perception of empowerment from leader humor is more likely to translate into bootlegging because their high trust in leadership lowers the risk assessment value of bootleg innovation. The results indicate that leadership emotional trust positively moderates the mediating role of psychological empowerment between leader humor and bootleg innovation behaviors. The higher the degree of emotional trust, the stronger the mediating role of psychological empowerment plays between leader humor and bootleg innovation behavior, and vice versa. Therefore, this study investigates the moderating role of leadership emotional trust in the influence process of “leader humor–psychological empowerment–bootlegging” and enriches the boundary conditions of the effect of leader humor at the level of interpersonal interactions.

### Managerial implications

The implications from this study are as follows:

(1) Companies should pay attention to the importance of leader humor, consciously cultivate and train the humor style of leaders, and improve the level of leader humor. In the daily interaction between employees and leaders, leader humor shows the more positive side and closes the psychological distance. Moreover, leaders reduce the hierarchical differences through such light-hearted communication as humorous words, thereby creating an equal working atmosphere. That is, this case makes the atmosphere in the organization more relaxed, increases their sense of belonging and intimacy, and improves employees’ sense of meaning, autonomy, and effectiveness at work. In today’s increasingly competitive marketplace, traditional mechanical empowerment management methods are becoming increasingly ineffective. In addition, employees’ perception of psychological empowerment is becoming increasingly important to organizational development. Leader humor can significantly provide employees with psychologically empowering perceptions, such as an increased sense of job meaning, job self-efficacy, and job autonomy. This case, in turn, can motivate employees to engage in behavior that benefits the organization. Therefore, leaders can significantly increase the level of psychological empowerment of their employees by using humor management strategies. As an empowerment tool and management strategy, leader humor helps enhance the management effectiveness of leaders.

(2) Leaders need to pay attention to the complexity of the impact of humor and make good use of the double-edged sword of leadership humor. Considering the benign violation properties of humor and the exemplary role of leaders, leader humor may have a mixed effect on employee behavior. The higher sense of psychological empowerment brought by leader humor, on the one hand, sends the signal that organizational norms can be easily violated and that organizational norms are less binding and prone to bootleg behavior. On the other hand, such a higher sense enhances employees’ pro-organizational behaviors, such as innovative behaviors. Leader humor brings higher psychological empowerment, ultimately producing a benign violations, such as bootlegging behavior, pro-organizational non-ethical behavior, and constructive bootleg behavior. Therefore, leaders should pay attention to the use of humor as a management tool, guide the positive side of humor, and avoid its negative side.

(3) Leaders look out for their employees, improve rapport, enhance credibility, and create a trustworthy managerial image. Leadership emotional trust has an important impact on the effectiveness of psychological empowerment. In view of the importance of psychological empowerment in the organization, leaders need to care about the wellbeing of employees and improve their credibility to make psychological empowerment more effective.

### Research limitations and future directions

The data in the questionnaire of this study are all based on the form of employee self-reporting. The common method bias is controlled and tested through the scientific design of the preliminary questionnaire and the later testing by statistical methods. However, common method bias problems, such as social approval, may still exist because of the hidden characteristics of the survey respondents and variables. In addition, this study does not separate the dimensions of psychological empowerment and compare the differences in mediation effects of each dimension, which has some deficiencies. In addition, this study reveals that leadership humor is an organizational factor to stimulate employees’ deviant innovation behavior from the micro perspective of organizational behavior, but it does not involve macro factors, which is an important research limitation. Especially since the outbreak of the COVID-19, employees’ psychology and behavior had been significantly affected, and this study did not consider the impact of the change of this important macro social environment factor on employees’ psychological empowerment and deviant innovation behavior.

Future research may consider expansion in terms of research design methodology and research content. The main focus of the study design methodology is to rationalize the study data acquisition, which can be optimized by using the method of multiple data sources (matching of leaders and employees). As the literature on the theme of leader humor is not yet available in China, the content of the study may consider further expanding the antecedents and consequences. There is still much room for expanding the influence effect, mechanism, and boundary conditions of leader humor. In terms of the influence effect, we can mainly focus on team-level outcome variables and study across levels. For the role mechanism, we can consider from the perspective of organizational (leadership) identity, emotion, and resources. For the regulation mechanism, we can consider the perspective of employee organizational (leadership) trait matching and the cross-Chinese and Western cultural perspectives. In addition, the macro social environment is also one of the important factors affecting employees’ deviant innovation. For example, we can explore the impact of the outbreak of COVID-19 on employees’ deviant innovation behavior in the future. According to the fear management theory, the death reminder caused by the COVID-19 will increase employees’ fear of death. In order to reduce the anxiety caused by fear of death, employees will engage in more constructive deviant behaviors that help improve the wellbeing of the organization to restore self-worth and meet self-esteem needs (Hu et al., 2020).

## Conclusion

Following the research logic of “leadership style–psychological motivation–employee behavior” and based on the BVT and the SCT, this study explored the influence mechanism of leader humor on bootlegging from the perspective of motivation. This study also complemented the research on the direct empowerment path between leader humor and employee behavior. The study helps enrich the research on the outcome variables of leader humor. This study also has an important practical value for using humor more effectively as an empowerment management tool for Chinese leaders to further enhance leadership effectiveness. The main findings of the study are as follows. (1) Leader humor has a positive impact on bootlegging. Leader humor is essentially a kind of benignant bootleg behavior, which will be imitated and studied by employees. They are the so-called people who follow the example of their superiors. (2) The mediating role of psychological empowerment between leader humor and bootlegging has been verified. Humor is a tool for empowerment. Leader humor creates a positive perceptions of empowerment for employees, such as increasing the sense of meaningfulness of their work and enhancing their perception of job autonomy and effectiveness. This high sense of empowerment is the cognitive basis for bootlegging. Stimulating the motivation to engage in risky bootlegging behavior is possible only after employees perceive high work meaning, high self-efficacy, and high work autonomy. Psychological empowerment provides psychological resources and conditions for bootlegging. (3) Leadership emotional trust positively moderates the impact of psychological empowerment on bootlegging and also strengthens the mediating role. A moderating mediating effect is observed. Leader humor can significantly promote the sense of psychological empowerment and enhance bootlegging, but employees can transform their psychological empowerment cognition into bootlegging, which is largely affected by the degree of leadership emotional trust. The effectiveness of leader humor is related to the leadership emotional trust.

## Data availability statement

The raw data supporting the conclusions of this article will be made available by the authors, without undue reservation.

## Author contributions

XZ: writing – original draft preparation, review, and editing. XS: conceptualization, and methodology. SM gave many constructive suggestions on theoretical construction and hypothesis deduction. CZ: formal analysis, investigation, and data curation. LM: visualization. All authors read and agreed to the published version of the manuscript, contributed to the article, and approved the submitted version.
